# An Analysis of Chemical Ingredients Network of Chinese Herbal Formulae for the Treatment of Coronary Heart Disease

**DOI:** 10.1371/journal.pone.0116441

**Published:** 2015-02-06

**Authors:** Fan Ding, Qianru Zhang, Carolina Oi Lam Ung, Yitao Wang, Yifan Han, Yuanjia Hu, Jin Qi

**Affiliations:** 1 State Key Laboratory of Quality Research in Chinese Medicine, Institute of Chinese Medical Sciences, University of Macau, Av. Padre Tomás Pereira S.J., Taipa, Macao 999078, China; 2 Pharmacy School, Zunyi Medical University, No.201 Dalian Road, Zunyi 563003, China; 3 Department of Applied Biology & Chemical Technology, The Hong Kong Polytechnic University, 11 Yuk Choi Road, Hung Hom, Kowloon, Hong Kong 999077, China; 4 Department of Complex Prescription of Traditional Chinese Medicine, China Pharmaceutical University, 639 Longmian Avenue, Nanjing 211198, China; University Heart Center Freiburg, GERMANY

## Abstract

As a complex system, the complicated interactions between chemical ingredients, as well as the potential rules of interactive associations among chemical ingredients of traditional Chinese herbal formulae are not yet fully understood by modern science. On the other hand, network analysis is emerging as a powerful approach focusing on processing complex interactive data. By employing network approach in selected Chinese herbal formulae for the treatment of coronary heart disease (CHD), this article aims to construct and analyze chemical ingredients network of herbal formulae, and provide candidate herbs, chemical constituents, and ingredient groups for further investigation. As a result, chemical ingredients network composed of 1588 ingredients from 36 herbs used in 8 core formulae for the treatment of CHD was produced based on combination associations in herbal formulae. In this network, 9 communities with relative dense internal connections are significantly associated with 14 kinds of chemical structures with P<0.001. Moreover, chemical structural fingerprints of network communities were detected, while specific centralities of chemical ingredients indicating different levels of importance in the network were also measured. Finally, several distinct herbs, chemical ingredients, and ingredient groups with essential position in the network or high centrality value are recommended for further pharmacology study in the context of new drug development.

## Introduction

Traditional Chinese Medicine (TCM) is the basis of health care in China for thousands of years. TCM uses herbal formulae to treat a wide variety of diseases including cancer [1], chronic diseases [2] and cardiovascular disease [3] based on a holistic approach with clinically-proven efficacy. Herbal formulae usually consist of multiple herbs which activate or synchronize each other when administered to help patients re-achieve the well balance of the body system [4]. Moreover, it is widely believed that the mechanism of multi-component and multi-target may be essential for herbal formulae to exert their clinical effects [1, 5–9]. However, a full understanding of this complex mechanism remains one of the biggest challenges in developing TCM [10].

In an attempt to resolve this problem, TCM studies usually follow a strategy of extraction, isolation, purification, structure identification, and pharmacological research of single or several herbal medicines. To date, more than 30,000 chemicals have been isolated form hundreds of Chinese herbal medicines, which are believed to be responsible for more than 120,000 kinds of pharmacological activities [3]. Vigorous effort in this research area has resulted in accumulation of immense amount of clinical and experimental data. The massive data volume displays a complex and nonlinear characteristic [5] which makes comprehensive analysis using conventional methods highly difficult. To date, few studies have been conducted to integrate and interpret the large volume of herbal data to achieve full understanding of the substance basis of TCM formulae.

In this context, this study attempts to explore a strategic method which allows comprehensive analysis of a massive amount of complex data to determine any underlying regularities and valuable information in TCM studies for drug development. According to the literature, the methods of information discovery in database have been suggested as promising approaches [11]. Among these approaches is network analysis [12–19], which has been widely applied to TCM for screening synergistic drug combination [20], establishing network pharmacology of TCM [21], uncovering formulae combination rules [22–23], and predicting drug targets [24]. Considering the advantages of visualization, readability and statistical function, network analysis was elected as the method of choice in this study.

To demonstrate the application of network analysis, Chinese herbal formulae for the treatment of one of the cardiovascular diseases (CVDs) were chosen as the target of analysis. CVDs are the leading cause of death in the world with a mortality rate estimated to reach 23.6 million by 2030 [25]. As the most common type of CVDs, coronary heart disease (CHD) is associated with high risks of hospitalization and death. The TCM formulae that have been used to treat CHD such as Compound Danshen Dripping Pills [26] and Shexiang Baoxin Pill [27] have been proven to have definite curative effects.

By applying network approach to selected Chinese herbal formulae for the treatment of CHD, the objectives of this study are to construct chemical ingredients network of herbal formulae based on combination associations of herbs, to analyze ingredients network in particular by detecting featured communities, and to provide a list of candidate herbs, chemical constituents, and ingredient groups for further pharmacology study in the context of new drug development.

## Methodology

### Data

In TCM, CHD are classified based on eight basic syndromes according to *2002 TCM Clinical Research Guidelines* issued by the China Food and Drug Administration. For the treatment of each syndrome in TCM clinical practice, physicians usually follow the most classic and accurate principle, which is “one formula for one specific syndrome” [28]. This study also followed this principle. Therefore, there were eight formulae being studied, each of which was specific to each of the eight basic syndromes of CHD. These eight formulae were also referred to as the core formulae. In practice, the formulae might undergo minor adjustments by adding or subtracting adjuvant herbs to meet the need of personalized medicines [29]. However, in order to avoid noises raised by adjusted formulae, the possibilities of minor adjustments of the formulae were not considered in this study. Information regarding the eight core formulae was retrieved from the traditional literatures in TCM [30]. Table 1 shows the eight syndromes, the eight formulae, and the related 36 herbs used as the study samples in the research.

Subsequently, all the reported chemical ingredients of each herb were determined with reference to Combined Chemical Dictionary (2009). Among the 36 herbs used in the 8 core formulae, 1588 chemical ingredients were identified. The chemical ingredients are not equally distributed in the 36 herbs with mean value being 44.78 (SD = 59.365), the maximum being 248, and the minimum being 2. These chemical ingredients were then categorized into groups based on their chemical structures indicated in SciFinder in accordance with the classification criteria suggested in Natural Pharmaceutical Chemistry and other literatures. To simplify the chemical structure classification system in this study, only the primary chemical structures were considered which numbered at a total of 14 chemical structural types. They were namely alkaloids, amino acid, fatty acid, flavonoids and its glycosides, glycosides, phenylpropanoids and its derivate, protein and enzyme, quinones, saccharides and its derivate, steroids and its glycosides, stilbenoids, terpenoids and its derivate, triterpenoids and its glycoside, and volatile oils.

**Table 1:** Sampled formulae and herbs.

Syndromes	Formulae	Herbs
Heart-blood stagnation	Xuefu Zhuyu Decoction	*Persicae Semen*, *Carthami Flos*, *Angelicae Sinensis Radix*, *Paeoniae Radix Rubra*, *Rehmanniae Radix*, *Chuanxiong Rhizoma*, *Aurantii Fructus*, *Platycodonis Radix*, *Bupleuri Radix*, *Achyranthis Bidentatae Radix*, *Glycyrrhizae Radix et Rhizoma*
Phlegm-blood stasis syndrome	Gualou Xiebai Banxia Decoction	*Trichosanthis Fructus*, *Allii Macrostemonis Bulbus*, *Pinelliae Rhizoma*
Qi deficiency and blood stagnation	Buyang Huanwu Decoction	*Astragali Radix*, *Angelicae Sinensis Radix*, *Paeoniae Radix Rubra*, *Pheretima*, *Chuanxiong Rhizoma*, *Carthami Flos*, *Persicae Semen*
Cold congelating	Guanxin Suhe Pills	*Styrax*, *Santali Albi Lignum*, *Aucklandiae Radix*, *Borneolum*, *Olibanum*
Yang deficiency of heart and kidney	Zhenwu Decoction	*Poria*, *Paeoniae Radix Alba*, *Atractylodis Macrocephalae Rhizoma*, *Zingiberis Rhizoma Recens*, *Aconiti Lateralis Radix Praeparata*
Yin deficiency of heart and kidney	Tianwang Buxin Dan	*Asparagi Radix*, *Ginseng Radix et Rhizoma*, *Poria*, *Scrophulariae Radix*, *Salviae Miltiorrhizae Radix et Rhizoma*, *Polygalae Radix*, *Platycodonis Radix*, *Angelicae Sinensis Radix*, *Schisandrae Chinensis Fructus*, *Ophiopogonis Radix*, *Platycladi Semen*, *Ziziphi Spinosae Semen*, *Rehmanniae Radix*
Qi-Yin deficiency	Shengmai San	*Ginseng Radix et Rhizoma*, *Ophiopogonis Radix*, *Schisandrae Chinensis Fructus*
Qi-blood stagnation	Huoluo Xiaolin Dan	*Angelicae Sinensis Radix*, *Salviae Miltiorrhizae Radix et Rhizoma*, *Olibanum*, *Myrrha*

### Network Construction

**Fig 1:**
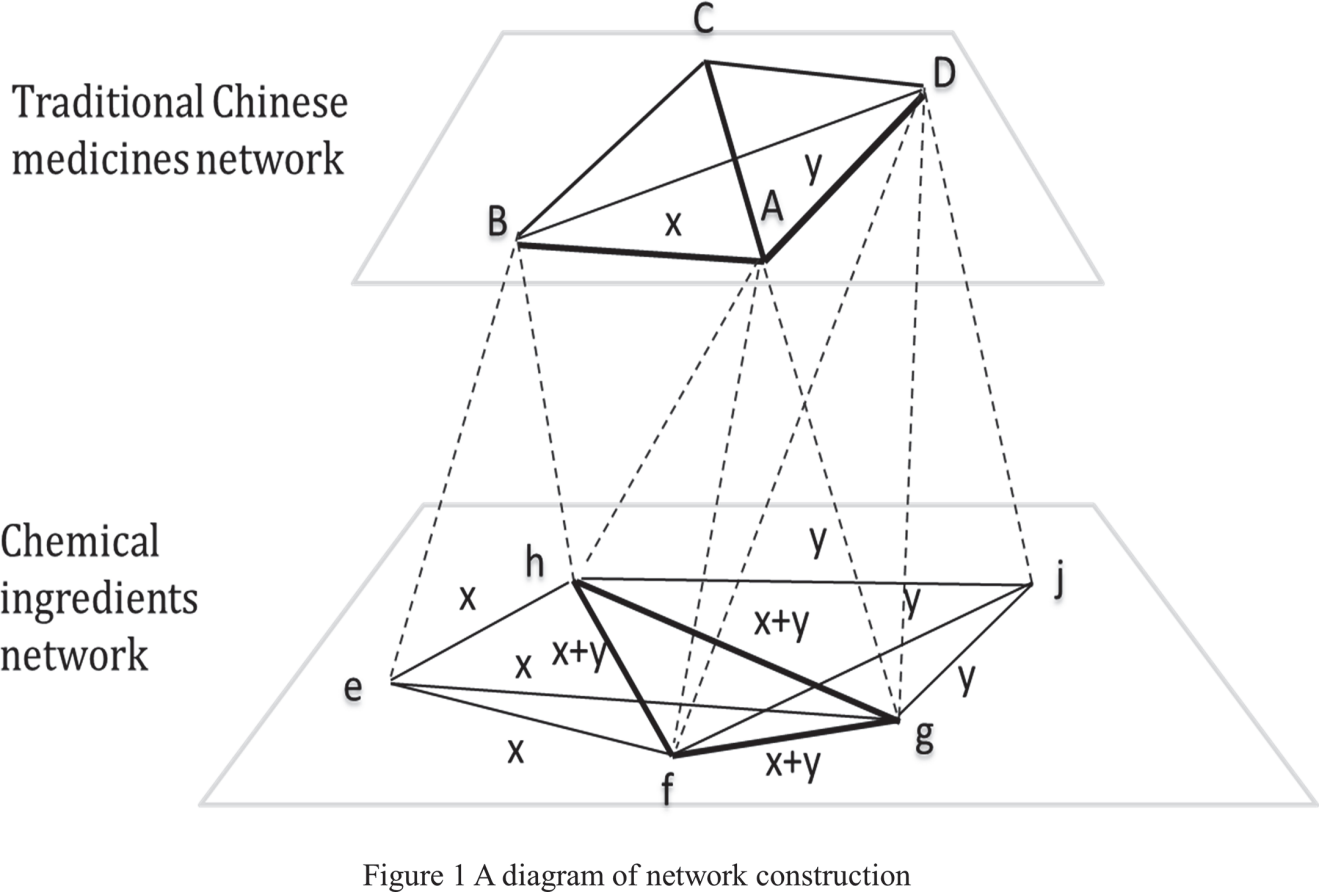
A diagram of network construction.

The complex relationships of herbs or chemical ingredients in Chinese herbal formulae can be described as networks. In the network, *V* is a set of vertices representing herbs or chemical ingredients, and *E* is a set of weighted edges where elements of *E* are unordered pairs of distinct vertices {*v*_*i*_, *v*_*j*_} as well as their weights representing the frequency of using pairs {*v*_*i*_, *v*_*j*_} in combination in sample formulae. A network *G* is the combination of *V* and *E*, *G* = (*V*, *E*). Herbal network *G*_*TCM*_ = (*V*_*TCM*_, *E*_*TCM*_) is constructed based on the combination frequency between herbs in selected sample formulae. Moreover, chemical ingredient network *G*_*CI*_ = (*V*_*CI*_, *E*_*CI*_) is produced by projecting *G*_*TCM*_ onto of chemical ingredients plane. Details are shown in Fig. 1.

The above figure was constructed to vividly present the projection process. The first layer network is traditional Chinese medicines network and the second layer is chemical ingredients network. The broken lines connecting two layers represent the projection process. Assuming *A*, *B*, *C* and *D* are the herbs in traditional Chinese medicines network, *A* have three chemical ingredients *h*, *f* and *g*, *B* have two chemical ingredients *h* and *e*, and *D* have three chemical ingredients *f*, *g*, *j*. It is assumed that all chemical ingredients from these two connected herbs have interactions with each other and obtain equivalent weights in herbal network. The projection of herb pair *AB* with the weight value of *x* generates six chemical ingredients pairs *ef*, *eg*, *fh*, *fg*, *gh*, and *eh*, all of which obtain same weight *x*. Similarly, herb pair *AD* weighted by *y* produces *hf*, *hg*, *hj*, *fg*, *fj*, and *gj*, all of which gain new weight *y*. In this way, any herbal pair can contribute to $C_{m+n-r}^{2}$ ingredient edges, where *m* and *n* are the number of chemical ingredients of the two herbs composing the herbal pair, and *r* represents the number of overlapping chemical ingredients that these two herbs have. Edges in herbal network are one by one projected onto chemical ingredients plane, while weights in ingredient network are accumulatively calculated correspondingly. In this diagram, the weight of edge *hf* is accumulatively counted as *x+y*, while weights of *fg* and *hg* are calculated similarly.

In this context, *E*_*CI*_ projected by *E*_*TCM*_ reflects combination associations of chemical ingredients in herbal formulae, because *E*_*TCM*_ originally records how many times the herbal pairs are used in combination in traditional formulae. Moreover, the chemical ingredients network was visualized using Gephi 0.8.2, which is an open source software project for visualization. This network offers a vivid means of uncovering the interaction of chemical ingredients and highlighting important chemical ingredients and ingredient groups.

### Network Analysis

Community identification and centrality measurement were employed in this study to analyze chemical ingredients network. One mesoscopic structure of network, which is called a community, consists of a group of nodes that are relatively densely connected to each other but sparsely connected to other dense groups in the network [31–33]. The method used for community detection was a heuristic method based on modularity optimization which was based on the maximization of an objective function [34–36]. In this study, chemical ingredients network is divided into several communities based on combination associations between chemical ingredients in herbal formulae.

On the other hand, centrality is a useful graph-theoretic approach to measure the importance of nodes. Four different types of centrality measures, namely degree centrality, closeness centrality, betweenness centrality and eigenvector centrality [37] were used in this study and calculated for each chemical ingredient in order to examine different aspects of network position. The former three types of centrality measures have been commonly used in network analysis. Besides, eigenvector centrality is also an important centrality that depends both on the number and the quality of its connections by examining all nodes in parallel and assigning centrality weights that correspond to the average centrality of all neighbors. A high eigenvector centrality of a node indicates that this node is connected with other nodes that also show many connections, rather than to peripheral nodes.

## Results

### Chemical Ingredients Network

**Fig 2:**
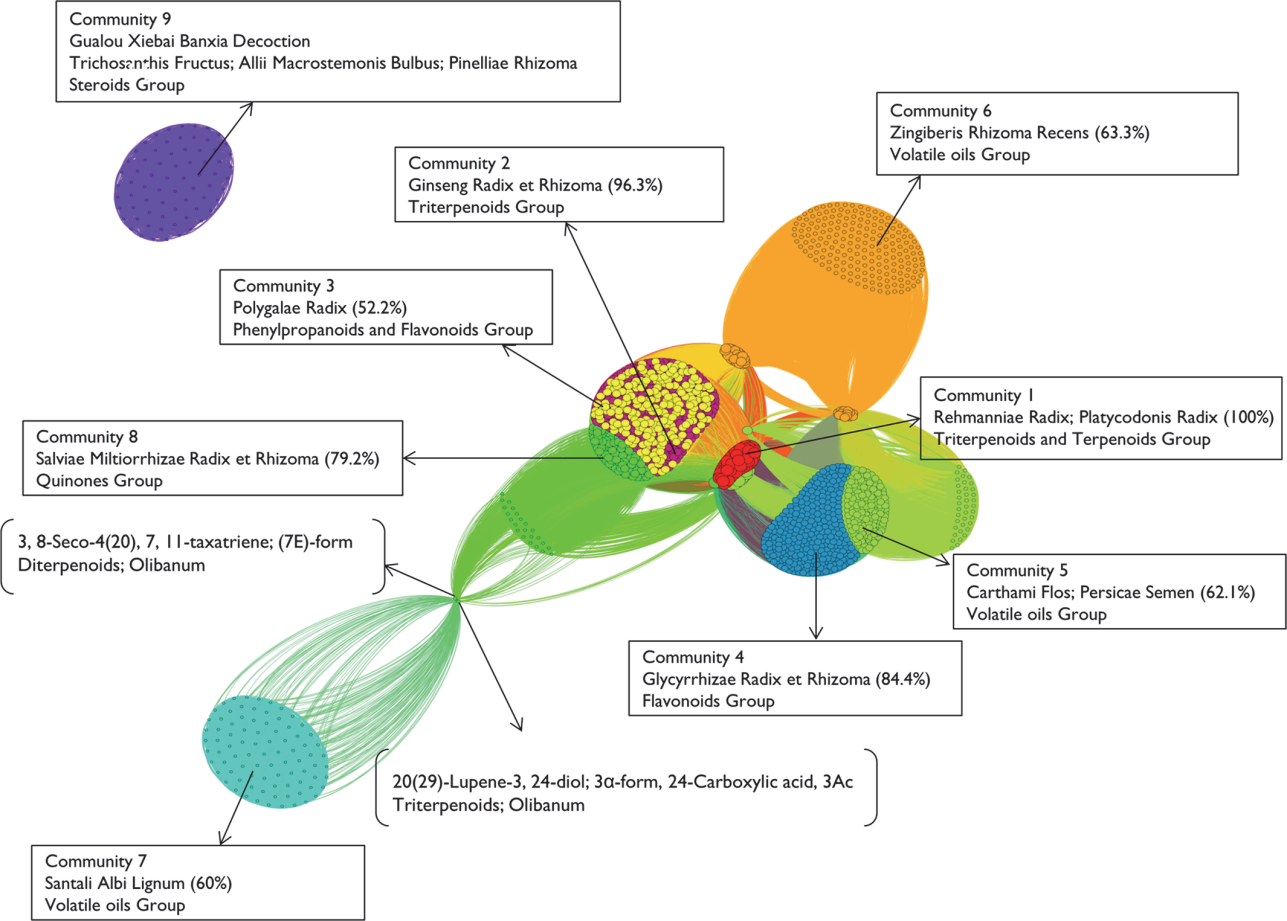
Chemical ingredients network. Note: Node represents chemical ingredient, while node size corresponds to the weighted degree centrality of a chemical ingredient. Each node is colored according to community membership, which was determined using a modularity optimization method. The strength of the lines corresponds to the frequency of the combination into sampled formulae between two chemical ingredients. Vertex positions determined using spectral graph analytic methods according to the normalized Laplacian so that chemical ingredients that are strongly interconnected positioned nearer to each other. Boxes illustrate outstanding nodes and show dominant structural types of chemical ingredients characterizing communities. The numbers in the parentheses in the boxes indicate how many percentages of chemical ingredients are contained by representative herbs of specific communities. Representative herbs are top one or two Chinese medicines which contain more than 50% chemical ingredients in specific communities. Square brackets specify chemical ingredients at the distant positions by showing their chemical names, chemical structural types, and corresponding herbs.

The chemical ingredients network is shown in Fig. 2. It was constructed based on the combination of chemical ingredients in sampled herbal formulae.

According to graph theory, the chemical ingredients network shown in Fig. 2 does not appear to be a random, meaningless outcome. Instead, it suggests some significant structural information especially when considering the notable nodes and relatively separated communities with dense internal connections. There are different dominant herbs and chemical types in different communities in chemical ingredients network. To certain extent, this network visualizes the substance basis of herbal formulae treating CHD based on combination rules among herbs. In terms of chemistry, the herbal formulae used to treat CHD are essentially mixtures of organic chemical ingredients. As shown in Fig. 2, communities are differentiated based on specific dominant herbs and chemical types according to combination associations of Chinese medicines. Based on the theory of structure-activity relationship, it may be significant to explore biological, functional or pharmacological implications of these communities with respect to herbal combination in further experimental studies.

### Community Analysis

The sampled 1588 ingredients were classified into 9 communities from the network-communities perspective based on combination frequency in the 8 core traditional formulae. Simultaneously, all the ingredients were also categorized into 14 groups based on their chemical structural types. In order to explore the association between network communities and chemical structural types, 9×14 Chi-square test between these two for the entire sampled ingredients was conducted. The result indicated that the communities in chemical ingredients network were significantly associated with chemical structural types with P<0.001 (χ^2^ = 2734.966 and N = 1588). It might be interpreted that chemical structural types had substantial influence on herbal combination.

**Fig 3:**
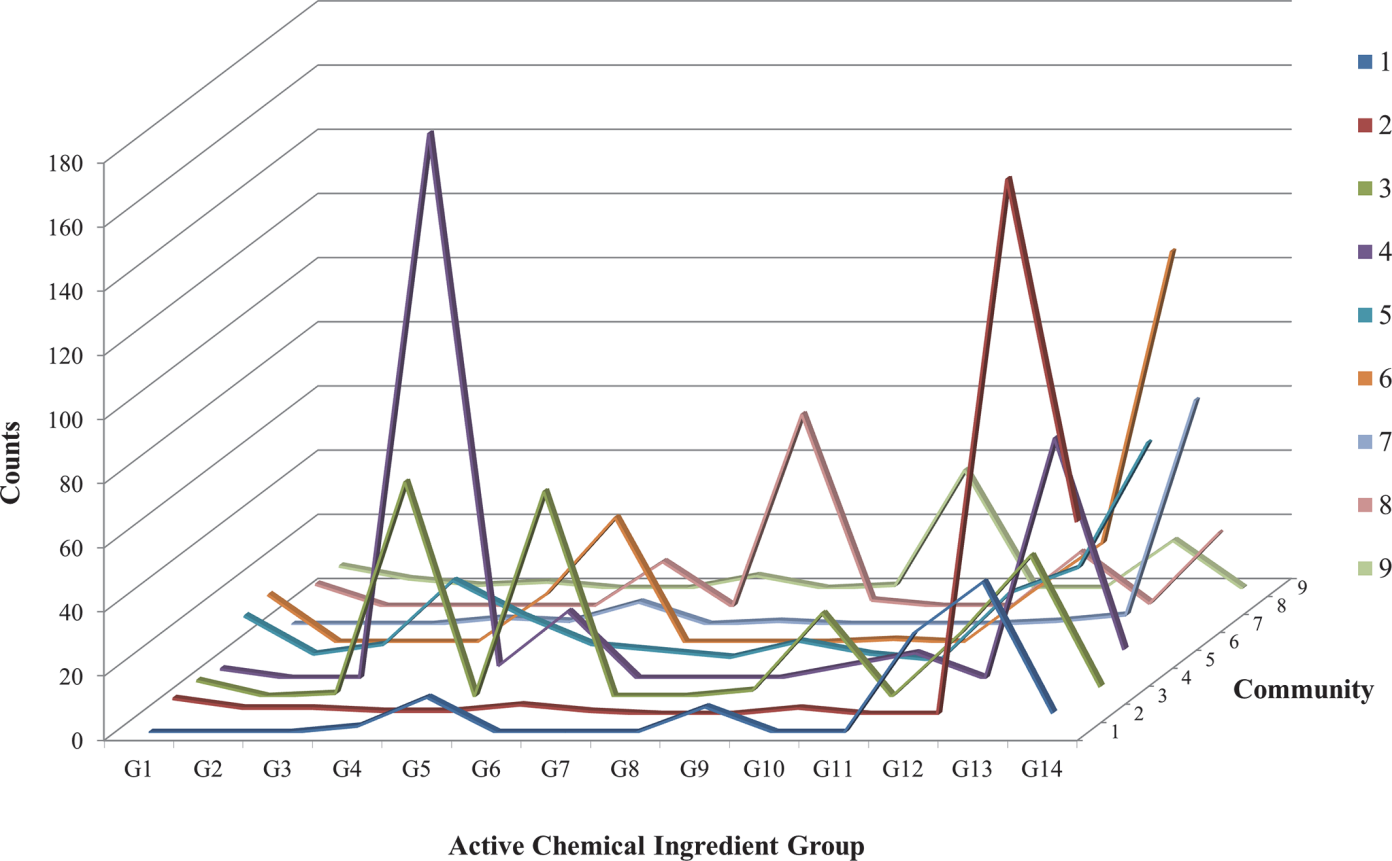
Chemical ingredients fingerprint. Note: G1 Alkaloids; G2 Amino acid; G3 Fatty acid; G4 Flavonoids and its glycosides; G5 Glycosides; G6 Phenylpropanoids and its derivates; G7 Protein and Enzyme; G8 Quinones; G9 Saccharides and its derivates; G10 Steroids and its glycosides; G11 Stilbenoids; G12 Terpenoids and its derivates; G13 Triterpenoids and its glycosides; G14 Volatile oils.

Fig. 3 suggests that it might worth elaborating the chemical structure profile of specific network communities in terms of the significant association among them, or in other words, detecting the chemical structural fingerprints of network communities. The graph clearly indicates that each community has its representative chemical ingredient groups. Specifically, triterpenoids and terpenoids are the major groups in community 1; community 2 is dominated by triterpenoids group; flavonoids and phenylpropanoids are almost equally dominant in community 3; flavonoids takes the major role community 4; community 5 consists of a wide variety of chemical ingredient groups with volatile oils taking up the highest percentage; volatile oils is also the major group in community 6; 82% of chemical ingredients in community 7 is volatile oils; quinone is the primary group in community 8; and community 9 is dominated by steroids group. These representative chemical ingredient groups specific to network communities are worth of further pharmacological examinations.

Among all these 14 chemical structural types, triterpenoids, volatile oils and flavonoids group are the most dominant, taking up 25.9%, 22.7% and 16.9% overall respectively. Correspondingly, herbs which have predominance from community 1 to community 9 are *Rehmanniae Radix* and *Platycodonis Radix*, *Ginseng Radix et Rhizoma*, *Polygalae Radix*, *Glycyrrhizae Radix et Rhizoma*, *Carthami Flos* and *Persicae Semen*, *Zingiberis Rhizoma Recens*, *Santali Albi Lignum*, *Salviae Miltiorrhizae Radix et Rhizoma*, and *Trichosanthis Fructus* and *Allii Macrostemonis Bulbus*, respectively.

Another important observation is in community 9 which appears to be an isolated entity in the chemical ingredients network. Community 9 composes of 70 active chemical ingredients originated entirely from three herbs, *Trichosanthis Fructus*, *Allii Macrostemonis Bulbus*, and *Pinelliae Rhizoma*. These 3 herbs constitute a classic herbal formula, Gualou Xiebai Banxia Decoction, for the treatment of CHD. Based on this finding, it is suggested that specific anti-CHD activities of chemical ingredients and ingredient groups in community 9, with the related steroids and its glycosides in particular which dominate in this community, may be good candidates for further investigation.

### Centrality Measures

**Fig 4:**
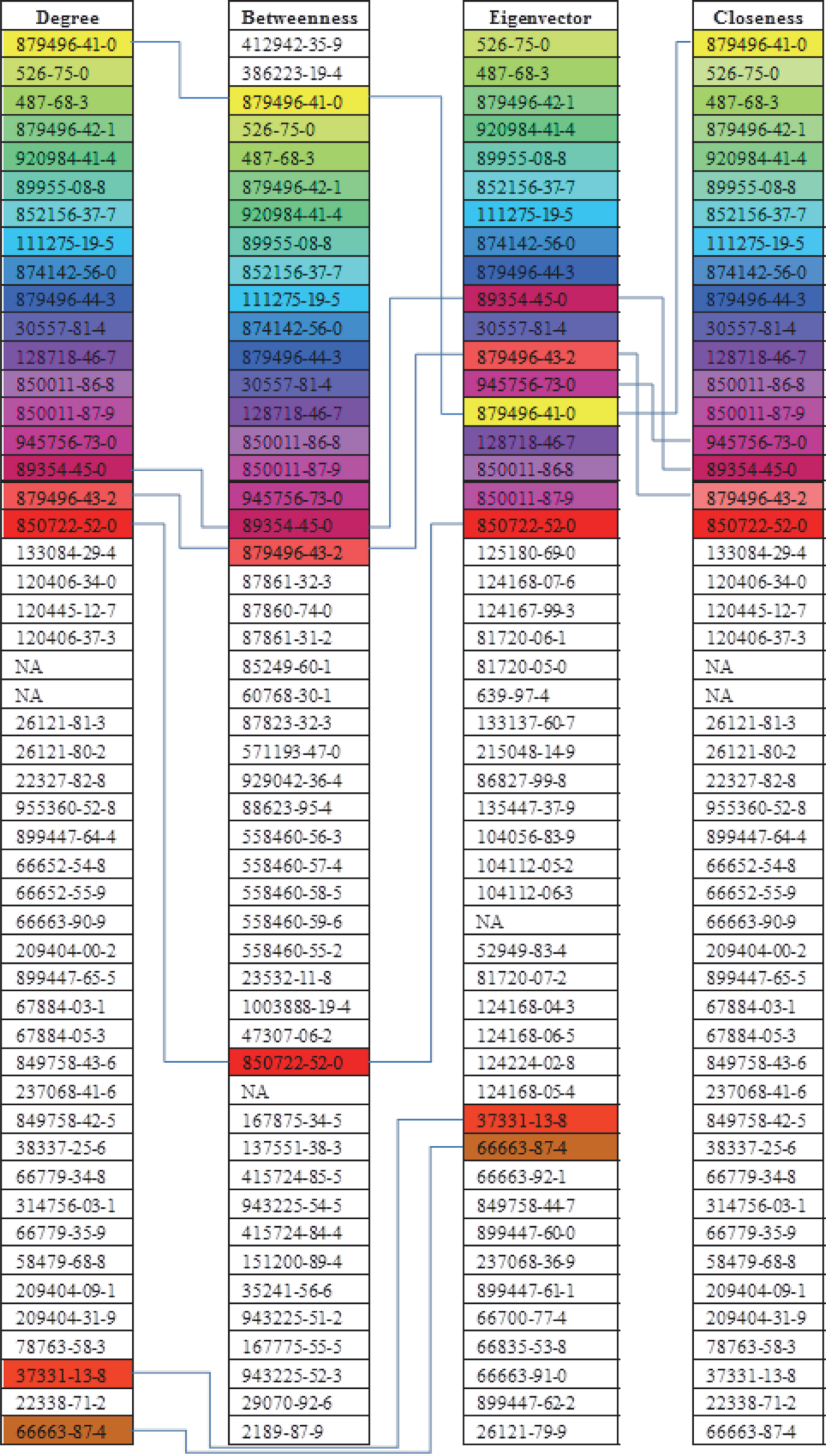
Ingredient ranking by different centralities. Note: The NA represents chemical ingredient whose number of CAS is unavailable in SCIfinder.

As mentioned in Methodology, this study employed centralities as the main network parameters to analyze the importance of every node. Fig. 4 shows the top 50 chemical ingredients named by the number of Chemical Abstracts Service (CAS), ranking from the top to the bottom by four kinds of centralities based on their importance in the network. The ingredients with higher ranking have more important roles in view of corresponding centrality measure. In order to highlight the change of different centrality rankings which represent different degree of importance in the network, most of the same ingredients among different centrality measures were colored with same color and all the other centrality-specific ingredients whitened, and elbow connector lines were used to outline the main differences of colored parts. By comparing the ingredient ordering by degree, betweenness, eigenvector and closeness centralities, it was observed that the measures by degree, betweenness, eigenvector and closeness centralities were relatively identical especially for the top 20 ingredients. From the perspective of network analysis, ingredients with top centralities were considered to have more important roles in chemical ingredients network based on herbal combination relationship. This might provide a meaningful hint for discovering effective anti-CHD chemicals. Furthermore, the understanding of chemical ingredients of different importance in the network measured by different centrality parameters, in particular the multi-centrality importance of colored part and specific-centrality significance of the whitened part, might shed light on drug development opportunity.

Moreover, drug investigation opportunities can also be addressed by observing chemical ingredients at the distant positions as shown in Fig. 2. Most of these ingredients are listed in Fig. 4 with high centrality value. For example, “20(29)-Lupene-3,24-diol; 3α-form, 24-Carboxylic acid, 3-Ac” (CAS: 412942-35-9) and “3,8-Seco-4(20),7,11-taxatriene; (7E)-form” (CAS: 386223-19-4) from Olibanum show the highest betweenness centrality, which connect community 7 and 8 as essential gatekeepers at the same time.

According to the combination regularity of herbs, some ingredients with high value of centrality locate in the key position in the network and connect to different communities. In this context, the corresponding herbs, *Rehmanniae Radix*, *Olibanum*, *Astragali Radix*, *Angelicae Sinensis Radix*, and *Schisandrae Chinensis Fructus*, are worth further investigation. For instance, *Angelicae Sinensis Radix* is an important herb which constitutes numerous volatile oils and phenylpropanoids, and is used to treat acute myocardial ischemia for its proved effect on replenishing blood and promoting blood circulation [38].

## Discussion

The full understanding of material basis remains the biggest bottleneck issue for the development of Chinese medicines. The material basis of traditional herbal formulae for the treatment of CHD is not clear yet, despite a prolonged history of use in Chinese clinical practices with significant efficacy as reported in classic literatures and modern pharmacological experiments. It is generally agreed that the beneficial effects of CHD herbal formulae are essentially generated by specific chemicals which are organically combined to work together according to herbal combination rules. This study demonstrated a strategy to visualize the underlying regularities of the chemical ingredients and their communities based on herbal combination frequency.

The chemical ingredients network which was obtained in the present study visualizes the interaction of the eight formulae material basis, and demonstrates degree and regularities of the mutual effect of substance basis which were categorized into nine significant communities in the network.

The Chi-square test result indicates the significant relationship between network communities and chemical structural types. There appears to be a chemical structure tendency to generate a community. Based on the theory of structure-activity relationship, each type of chemical structure possesses a specific biological activity. For example, triterpenoids have numerous unique and potentially usable biological activities. They are studied for their anti-inflammatory, hepatoprotective and tonic effects. Considerable evidence implicates that the inflammatory pathway is significantly important in the CHD process [39]. However, there is disadvantage of using triterpenoids due to the toxicity associated with their haemolytic and cytostatic properties [40]. Possibly, the synthetic interactions with other types of chemical ingredients in the community might generate efficacy-enhancing and toxicity-reducing effects. In the present study, take community 2 for instance, triterpenoids take up 68 percent and volatile oil, which have anti-inflammatory activities, take up 25 percent of all the chemical ingredients. The data from community 2 suggest that pharmacological activities of this community are likely to be anti-inflammatory as its prime efficacy in the CHD treatment.

Moreover, the important ingredients, ingredient groups, and herbs identified in this study are worthy of further pharmacology investigation. For example, two molecules (CAS: 412942-35-9 and 386223-19-4) from Olibanum are distinct in the chemical ingredients network by connecting two main network communities, 7 and 8, which are composed of volatile oils and quinones, respectively. Given the close relationship between chemical structure and biological activity, ingredient groups in communities 7 and 8 may have different effects on the treatment of CHD, and are further synthesized to bring about overall therapeutic efficacy by the mediation of the two chemical constitutes from Olibanum. In this sense, this kind of virtual network-based screening of potential active ingredients in herbal formulae may generate various research hypotheses for pharmacological experiments. Meanwhile, exploration of the therapeutic mechanisms of traditional herbal formulae at the molecular level is of great significance to elucidate the scientific rationale and implications of TCM.

This research is also a valuable tool in the sense of translational medicine. Translational medicine offers a new medical research direction by filling in the current serious gaps between basic research and clinical practices [41]. Chinese herbal formulae are effective traditional solutions to treat diseases, and have been developed based on thousands of years of experience in clinical practice. However, until now, basic research in this field has not provided enough scientific evidence on the material basis and therapeutic mechanisms at the molecular level. This greatly limits the combination of TCM with Western drugs to form an integrative/alternative system of medicine for the treatment of complex diseases. This research translates clinical experience with herbal formulae for the treatment of CHD into a chemical ingredients network, where various potential candidates for pharmacology investigation are identified. This research, and subsequent experimental work in this regard, will be helpful to narrow the gap between fundamental and clinical research in TCM.

Finally, this research may be developed into an innovative approach to discover combination drug treatment to combat the limitations of the current single-target, single-compound paradigm when treating complex disease such as CHD. There is no doubt that the chance of finding a new drug candidate, which is a simple compound with super strong pharmacological activity derived from living organisms or herbs such as Artemisine through comprehensive screening and phytochemistry approaches, seems to be slimmer than ever. “Dirty drug” with multiple components may be an alternative path to new drug discovery in the future [42]. For instance, it is believed that the effects of the herbal formulae on CHD are not triggered by some compounds working individually. Instead, the effectiveness may result from the synergizing mechanisms of actions of a large number of chemical ingredients in the formulae, any of which may only have rather weak effect on CHD if acted individually. It appears to be a new and promising pathway to drug discovery which will give rise to a group of chemical ingredients each with specific chemical structure types. Moreover, the results also showed that the network-based approach has potential to make sense of the integrated effect of TCM formulae.

This study might be considered as the preliminary contribution to a new drug development pathway through mapping the Chinese herbal formulae/chemical ingredients combination network, featuring ingredient communities with tight internal combinations, and highlighting chemical ingredients with leading network centralities. The limitations encountered in this study included the lack of detailed pharmacological experiments on potential chemical ingredients and the related chemical groups for verification of the findings. In addition, in order to simplify the research questions, the dosage of herbs in the formulae were not taken into account. These areas are worth looking into in future research work as a continuation of this current study.

## Supporting Information

S1 Dataset Original data. (XLSX)
